# Coordinated Regulation of Anthocyanin Biosynthesis Genes Confers Varied Phenotypic and Spatial-Temporal Anthocyanin Accumulation in Radish (*Raphanus sativus* L.)

**DOI:** 10.3389/fpls.2017.01243

**Published:** 2017-07-19

**Authors:** Everlyne M'mbone Muleke, Lianxue Fan, Yan Wang, Liang Xu, Xianwen Zhu, Wei Zhang, Yang Cao, Benard K. Karanja, Liwang Liu

**Affiliations:** ^1^National Key Laboratory of Crop Genetics and Germplasm Enhancement, Key Laboratory of Horticultural Crop Biology and Genetic Improvement (East China) of MOA, College of Horticulture, Nanjing Agricultural University Nanjing, China; ^2^Department of Plant Sciences, North Dakota State University Fargo, ND, United States

**Keywords:** anthocyanin, color variation, coordinated regulation, gene expression, *Raphanus sativus*, spatial-temporal, transcriptome

## Abstract

Anthocyanins are natural pigments that have important functions in plant growth and development. Radish taproots are rich in anthocyanins which confer different taproot colors and are potentially beneficial to human health. The crop differentially accumulates anthocyanin during various stages of growth, yet molecular mechanisms underlying this differential anthocyanin accumulation remains unknown. In the present study, transcriptome analysis was used to concisely identify putative genes involved in anthocyanin biosynthesis in radish. Spatial-temporal transcript expressions were then profiled in four color variant radish cultivars. From the total transcript sequences obtained through illumina sequencing, 102 assembled unigenes, and 20 candidate genes were identified to be involved in anthocyanin biosynthesis. Fifteen genomic sequences were isolated and sequenced from radish taproot. The length of these sequences was between 900 and 1,579 bp, and the unigene coverage to all of the corresponding cloned sequences was more than 93%. Gene structure analysis revealed that *RsF3*′*H* is intronless and anthocyanin biosynthesis genes (ABGs) bear asymmetrical exons, except *RsSAM*. Anthocyanin accumulation showed a gradual increase in the leaf of the red radish and the taproot of colored cultivars during development, with a rapid increase at 30 days after sowing (DAS), and the highest content at maturity. Spatial-temporal transcriptional analysis of 14 genes revealed detectable expressions of 12 ABGs in various tissues at different growth levels. The investigation of anthocyanin accumulation and gene expression in four color variant radish cultivars, at different stages of development, indicated that total anthocyanin correlated with transcript levels of ABGs, particularly *RsUFGT, RsF3H, RsANS, RsCHS3* and *RsF3*′*H1*. Our results suggest that these candidate genes play key roles in phenotypic and spatial-temporal anthocyanin accumulation in radish through coordinated regulation and the major control point in anthocyanin biosynthesis in radish is *RsUFGT*. The present findings lend invaluable insights into anthocyanin biosynthesis and may facilitate genetic manipulation for enhanced anthocyanin content in radish.

## Introduction

Anthocyanins are a big group of naturally occurring water-soluble pigments that belong to a larger group of ubiquitous secondary metabolites referred to as flavonoids (Rodriguez-Saona et al., 1999). They play integral biological functions in plants by protecting plant tissues or senescing leaves against extreme temperatures, photo-oxidative injury and irradiation (Nhukarume et al., 2010). They also contribute in pollination and facilitating seed distribution (Harborne and Williams, 2000). Besides being directly beneficial to plants, anthocyanins have also been shown to display vital nutraceutical properties that prevent heart disease and cancer in humans (Lamy et al., 2007; Nhukarume et al., 2010). Additionally, red radish derived anthocyanins continue to be largely used in food industries as coloring agents because they are highly stable and exhibit properties similar to those of synthetic food Red No.40 (Rodriguez-Saona et al., 1999).

Anthocyanins are synthesized from the phenylpropanoid pathway comprised of multienzymes that catalyze several key biosynthetic steps (Figure S1; Park et al., 2011; Wei et al., 2011). The synthesis of anthocyanins is dependent on the enzymes of the general flavonoid pathway as well as those of the specific anthocyanin pathway. The enzymes which catalyze specific steps of the anthocyanin biosynthesis pathway are encoded by structural genes which are in-turn under the control of regulatory genes (transcription factors). In addition, substrate specificity for the genes between different leucoanthocyanidins with regard to the hydroxylation ring is a mechanisms that explains the variation in anthocyanin aglycons in different genotypes. The anthocyanin biosynthetic pathway is one of the earliest studied pathways since 1980 (Holton and Cornish, 1995). In various plant species most enzymes of the anthocyanin biosynthetic pathway are already identified (He et al., 2010; Park et al., 2011; Jaakola, 2013). The first stage of the general phenylpropanoid pathway is where phenylalanine is converted to coumarate-CoA by phenylalanine ammonia lyase gene (*PAL*), cinnamate 4-hydroxylase gene (*C4H*), and 4-coumarate CoA ligase gene (*4CL*). The anthocyanin pathway branches from the general phenylpropanoid pathway when enzymes chalcone synthase (*CHS*), chalcone isomerase (*CHI*), flavanone 3-hydroxylase (*F3H*) and flavonoid 3′-hydroxylase (*F3*′*H*), catalyze the synthesis of tetrahydroxychalcone (THC) from the combination of a 4-coumaroyl CoA molecule and three of malonyl-CoA (Tanaka et al., 2008). When *CHS* gene was modulated through RNA interference, the blue color of the flower changed from blue to white (Fukusaki et al., 2004). The flavonoid genes were also found to exhibit up-regulation with advance toward ripening stage, resulting in color development in strawberry (*F. ananassa*), which establishes a positive correlation between transcript levels of flavonoid genes and anthocyanin accumulation (Carbone et al., 2009). The immediate step is the generation of various anthocyanidins by dihydroflavonols, catalyzed by anthocyanidin synthase gene (*ANS*) and dihydroflavonol 4-reductase gene (*DFR*) which uses NADPH as a cofactor (Lepiniec et al., 2006). In *Pyrus pyrifolia* the *DFR* and *ANS* genes have been considered the limiting factors for the skin color of the mildly colored pears (Zhang et al., 2011). Over 20 types of, anthocyanidins/ aglycons, of anthocyanins are known (Jaakola, 2013), and are clustered into six major classes: cyanidin, delphinidins, malvidin, pelargonidin, peonidin and petunidin; the major anthocyanin aglycons in radish being cyanidin and pelargonidin (Park et al., 2011). The synthesized anthocyanidins then undergo modification through a series of methylation and glycosylation steps to form stable anthocyanidins. These steps are catalyzed by glucosyltransferases, glycosyltransferases and methyltransferases, which are encoded by a large number of genes depending on the anthocyanin aglycon and the genotype. In grape berry, it was reported that loss of color in white grapes was due to the absence of *UDP-glucose: flavonoid 3-O-glucosyltransferase* (*UFGT*) gene which is deemed critical for anthocyanin biosynthesis (Kobayashi et al., 2001). The downstream steps involve the mutual sequestration of the anthocyanins into the vacuoles, which involve the non-covalent activity of *glutathione S-transferase* (*GST*) genes. Several *GSTs* involved in the sequestration of anthocyanins have been isolated in several plants including apple (*Malus domestica*), Arabidopsis (*A. thaliana*) and grape (*V. vinifera*) (Cutanda-Perez et al., 2009; Li et al., 2011; Ahn and Yun, 2016). Although different genes may encode different families of GSTs, they are all necessary for the sequestration of anthocyanins into the vacuoles.

Radish (*Raphanus sativus*) is a member of the Brassicaceae family and among the most economically important root vegetable crops grown globally. The color of the taproots varies from white to red, to purple-pink, to green, to bicolor, due to its accumulation of large amounts of anthocyanins (Chen et al., 2016). Studies on anthocyanin and the underlying molecular mechanism in radish dwell on single-tissue analysis and mostly at maturity stage (Park et al., 2011; Bae et al., 2012; Chen et al., 2016). A growing body of research provides evidence that as growth advances, anthocyanin accumulation is not localized in the plant but rather, discriminatively accumulates in specific clusters of cells located in different plant tissues (Zuluaga et al., 2008; Zhang et al., 2014). Radish is rich in anthocyanin and a very versatile crop with regards to anthocyanin accumulation and distribution; while some varieties concentrate anthocyanins in the flesh, some accumulate in the skin, stems, leaves or both, depending on the genotype and developmental stage. These presents radish as an appropriate model organism for deciphering the mechanisms that contribute to differential anthocyanin accumulation.

Recently, several studies tried to elucidate the mechanisms that contribute to color variation in intra-tissues during different levels of growth. These include cherry (Liu et al., 2013), dendrobium (Kriangphan et al., 2015), mulberry (Li et al., 2014), grape (Xie et al., 2015) and yam (Yin et al., 2015). It was suggested that patterns of anthocyanin biosynthesis in different grape berry tissues are discontinuous, implying that ABGs are regulated spatially and temporally (Falginella et al., 2012; Xie et al., 2015). Transcript levels of anthocyanin biosynthetic genes in strawberry fruits were also found to be increased during fruit ripening with the expression levels being much higher in red colored tissues than white tissues (Salvatierra et al., 2010). In purple yam, anthocyanin biosynthesis genes were highly expressed at the early stages of growth in leaves and stems, but peaked at growth stages: the middle and later stages of growth (Yin et al., 2015). Radish differentially accumulates anthocyanin during various stages of growth, yet, molecular mechanisms underlying this differential anthocyanin accumulation remained unknown.

In this study, high-throughput sequencing data was employed to concisely identify key ABGs in radish. Secondly, four different radish cultivars were utilized to provide a comprehensive comparative spatial-temporal transcript analysis of candidate ABGs. Profiling at different growth stages also aimed at determining the stage at which the anthocyanin biosynthetic pathway switches off leading to the loss of anthocyanin in the non-colored radish. These findings could provide additional vital fundamental knowledge to dissect molecular mechanisms underlying differential anthocyanin accumulation and contribute to the ultimate genetic improvement of anthocyanin in radish taproots.

## Materials and methods

### Plant materials

**Figure 1:**
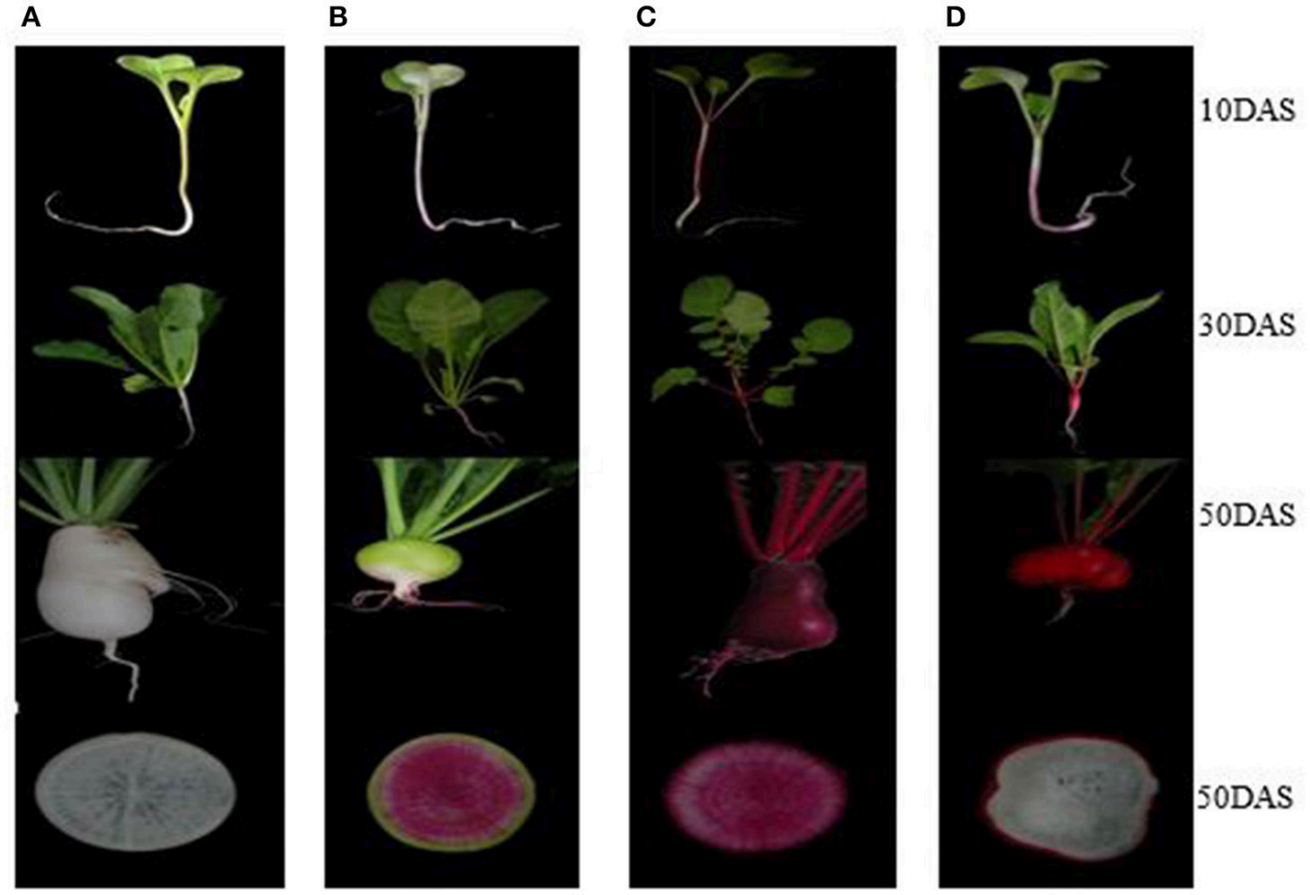
Four different colored radish genotypes at different developmental stages. **(A)** “NAU-XBC”, **(B)** “NAU-XLM”, **(C)** “NAU-YZH”, **(D)** “NAU-YH”.

Four advanced inbred radish lines “NAU-YH”, “NAU-XLM”, “NAU-XBC”, and “NAU-YZH” were used. The cultivars exhibit red skin-white flesh, green skin-pink-purple flesh, white skin-white flesh and red skin-red flesh, respectively (Figure 1). Seeds were selected and surface sterilized before being germinated on moist filter paper in darkness for 3 days. They were then transplanted into plastic pots containing 1:1 mixture of sterilized soil and peat substrate, and cultured in the greenhouse. The growth conditions included a 14 h light/10 h darkness photoperiod with an average temperature of 18°C. The inability of radish cortex cells to undergo division and expansion results in splitting, an occurrence that is vital for the initiation of taproot thickening. The development of cortex splitting is an important signal of the initiation of taproot thickening growth in radish due to the inability of the cortex cells to undergo division and expansion (Wang et al., 2013). Cortex splitting has been found to begin at around 12 days after sowing (DAS), thus 10 DAS is the pre-cortex splitting, while the peak of root cortex splitting is at 30 DAS. The maximum taproot thickening is achieved at 50 DAS. Subsamples of leaf and taproot issues were collected at 10 DAS (pre-cortex splitting stage), 30 DAS (cortex splitting stage) and 50 DAS (taproot thickening stage). At maturity stage, prior to experiments, radishes were briefly, manually peeled to separate the skin and the flesh, which were then cut into small cubes. Samples in three biological replicates were separated into different batches for anthocyanin and total RNA extraction. Samples for anthocyanin were used immediately, while those for RNA extraction were frozen in liquid nitrogen and stored at −80°C until use.

### Determination of total anthocyanin accumulation

Individual samples were ground into fine powder in the presence of liquid nitrogen before anthocyanin extraction. The anthocyanin content was measured with the modified method (Mehrtens et al., 2005; Yin et al., 2015). Totally, 2.00 g of the ground powder were dissolved in 6 mL of an extraction solution [concentrated HCl + 80% (v/v) ethanol, 1:99], then extracted by shaking on a mechanical shaker at 110 rpm at room temperature, while shielding from light for 24 h. Then 1 mL of the mature radish extract or 2 mL of seedling (10 DAS) extract was filtered and diluted to 10 mL with 0.4 M sodium acetate (pH 4.5) and 0.025 M hydrochloric acid (pH 1.0) buffers. The absorbance was then observed at 530 and 657 nm on a UV-VIS spectrophotometer (ND752, SPSIC, Shanghai, China). The anthocyanin content (Q) detected was calculated as Q $=\frac{A530\;-\;\left(0.25\times A657\right)}{\text{g FW}},$ where g FW is the fresh mass of the sample in grams. Values, representing means from three independent experiments were subjected to analysis of variance (ANOVA) using PROC GLM code of SAS version 9 (2005). Significant means were separated using the Tukey's Honestly Significant Difference Test at *P* ≤ 0.05, while Microsoft Office Excel version 2013 was used to generate figures.

### Genomic DNA, total RNA extraction and reverse transcription

The cetyltrimethylammonium bromide CTAB method (Liu et al., 2003) was used for extraction of genomic DNA from mature leaf and root tissues of “NAU-YH”. After being digested with RNase, it was re-extracted with phenol/chloroform/isoamyl alcohol (25:24:1) and chloroform/isoamyl alcohol (24:1). Total RNA from the leaf and root tissues of all the four cultivars at the three previously mentioned developmental stages was extracted according to the previous protocol (Xu et al., 2013). Integrity analysis of DNA and RNA was performed by electrophoresis on a 1% ethidium bromide stained agarose with gel. cDNA was synthesized from 2 μg of RNA using a PrimeScript™ RT reagent Kit with gDNA Eraser (TaKaRa Bio Inc., Dalian, China). Prior to reverse transcription, DNase was used to remove contaminating DNA according to the manufacturer's instructions. Total RNA was reverse-transcribed using M-MLV reverse transcriptase (Promega, Madison, WI, USA) and an oligo d (T) 18 primer.

### Identification, isolation, and sequence analysis of candidate anthocyanin biosynthesis genes (ABGs)

For RNA Sequencing Library Construction and Illumina Sequencing, radish (*Raphanus sativus* L.) advanced inbred line “NAU-YH” was used. Sequencing was done on Illumina HiSeq™ 2500 platform at the Beijing Genomics Institute (BGI, Shenzhen, China). The construction of the library and Illumina sequencing were performed according to a method previously described (Cheng et al., 2013). Radish unigene sequences from the *de novo* transcriptome data, deposited in the NCBI Sequence Read Archive repository: SRX707630 (Yu et al., 2016) were analyzed to identify and isolate the genes associated with anthocyanin biosynthesis. Using nucleotide sequences from these genes as queries, BLASTx was done against the NCBI Gene Bank (http://www.ncbi.nlm.nih.gov/genbank), radish genome (http://www.nodai-genome-d.org/) (Kitashiba et al., 2014) and “NAU-YH” transcriptome databases. For validation of the transcriptome data, fifteen candidate genes including *RsPAL1, Rs4CL3, RsCHS3, RsCHI, RsF3H, RsF3*′*H1, RsDFR, RsANS, RsANR, RsUFGT, RsTT12, RsSAM, RsOMT, RsGSTU5*, and *RsGSTF10* were isolated from “NAU-YH”. Gene-specific primers (GSPs) were designed based on the genomic nucleotides and used to amplify the ABGs using mixed root and leaf DNA as the template. Primers used for cloning are shown in Table S1. Each PCR reaction was carried out in a total volume of 20 μL containing 2.0 mM Mg^2+^, 0.2 mM dNTP, 1.0 μM of gene specific primer, 0.8 μL Taq DNA polymerase (TaKaRa Bio Inc., Dalian, China) and 20 ng of diluted DNA template. The conditions for PCR were as follows: 94°C for 3 min; 35 cycles of 50 s at 94°C, 50 s at annealing temperature (Tm) and 90 s at 72°C, and finally extension at 72°C for 10 min. PCR products were purified and cloned into a pMD19-T vector (TaKaRa Bio Inc., Dalian, China). Three independent positive clones from each isolated gene were sequenced on an ABI3730 sequencer (Applied Bio systems, USA). These cloned sequences were aligned with the corresponding unigenes from the transcriptome (https://www.ncbi.nlm.nih.gov/sra/SRX707630/), to access the coverage. The chromosomal location and related predicted sequences were obtained through a manual search from (http://www.nodai-genome-d.org/). ORF Finder and the BLAST (https://www.ncbi.nlm.nih.gov/orffinder/) programs were employed to analyze the cDNA and amino acid sequences. Pfam (http://www.pfam.xfam.org/) was used to identify conserved domains. Coding sequences (CDS) were aligned to DNA sequences and schematics generated using Gene Structure Display Server (http://gsds.cbi.pku.edu.cn/).

### Transcript profiling of anthocyanin biosynthetic genes (ABGs)

Following gene validation, a spatiotemporal analysis of the respective anthocyanin biosynthetic genes' transcript levels, was performed using quantitative real time PCR (RT-qPCR). Sequence-specific primers used for RT-qPCR were designed using Beacon Designer v7.0 (Premier Biosoft International, USA; Table S2). An iQ™ 5 Multicolor Real-Time PCR Detection System (Bio-Rad Laboratories, Berkeley, CA, USA) was used to perform PCR. Each reaction (20 μL) contained 10 μL of SYBR® Premix Ex Taq (Takara), 2.0 μL of cDNA and 0.2 μM of each primer. PCR was carried out under the program of 95°C for 3 min, 40 cycles of 95°C for 5 s, 58°C for 30 s, and 72°C for 10 s. The data were analyzed using iQ™ 5 Optical System Software (version 2.1, Bio-Rad) and expression levels of the gene normalized to *RsActin* gene (Xu et al., 2012). Relative fold expression changes were calculated using the 2^−ΔΔ*C*_T_^ method (Livak and Schmittgen, 2001). The ANOVA was performed using PROC GLM code of SAS version 9 (2005) and means separated using Tukey's Honestly Significant Difference Test at *P* ≤ 0.05, while Microsoft Office Excel version 2013 was used to generate figures. Specifically, point analysis was done, depicting the significant variations between the leaf and root tissue, within each gene. Pearson's correlation coefficient, MetaboAnalyst 3.0 (http://www.metaboanalyst.ca/faces/Secure/upload/StatUpload View.xhtml) was used for correlation analysis between gene expression levels and accumulation of total anthocyanin.

## Results

### Profiling of total anthocyanin in radish

**Figure 2:**
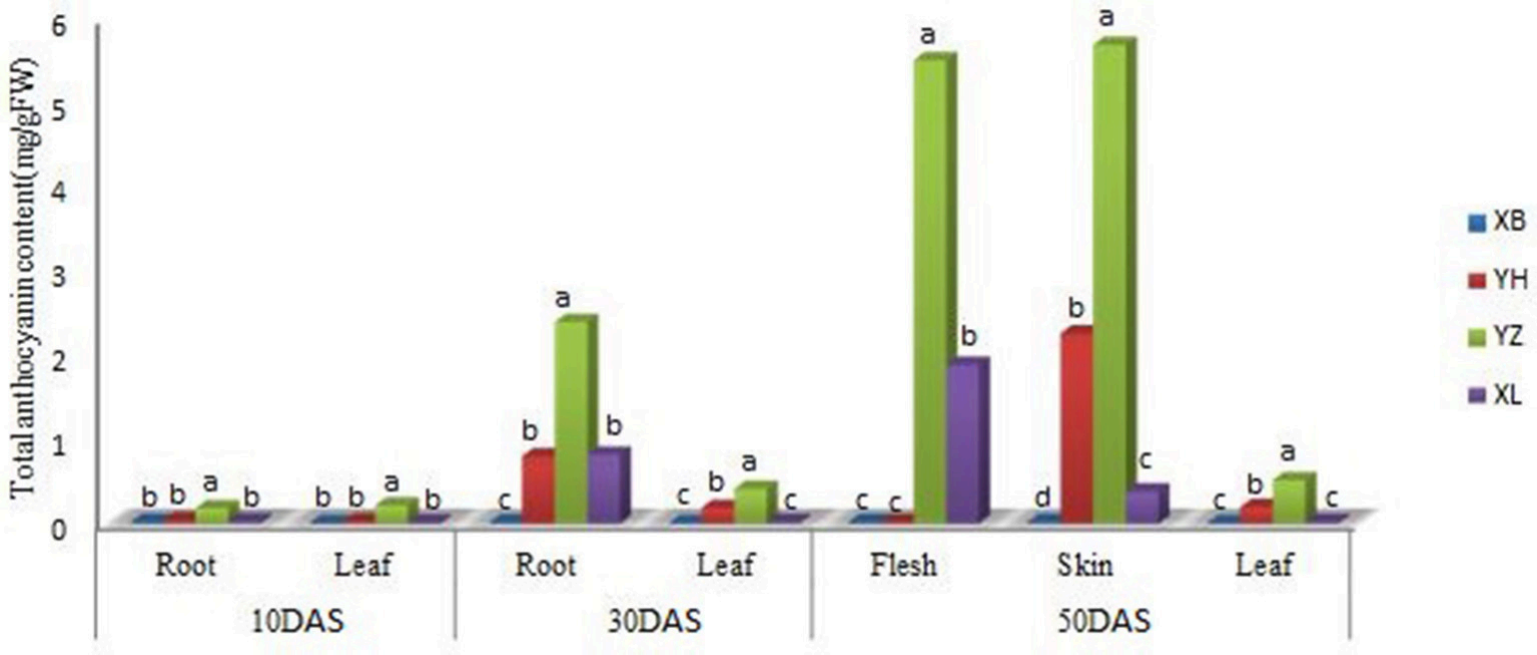
Changes in the concentration of anthocyanin (mg/g Fw) in the leaf and root of four radish cultivars during different stages of growth. XB, YH, YZ and XL represents “NAU-XBC”, “NAU-YH”, “NAU-YZH”, and “NAU-XLM”, respectively. Values are means of three independent replicates. Values not connected by the same letter within the same data point are significantly different according to Tukeys HSD test (*P* ≥ 0.5).

Differences in the quantity of anthocyanin were observed in the leaf, young root, flesh and skin of different radish genotypes at different growth stages (Figure 2). At all sampling points, there were no detectable amounts of anthocyanin in the leaf and root tissues of “NAU-XBC”. At 10 DAS significant anthocyanin amounts were only detected in the leaf and root of “NAU-YZH”. There was a steady global increase in anthocyanin content at 30 DAS, wherein, anthocyanin was detected in the leaves of “NAU-YH” and “NAU-YZH”, being highest in “NAU-YZH” while insignificant in “NAU-XLM”. In the root tissues at 30 DAS, anthocyanin was detected in “NAU-YZH”, “NAU-YH”, and “NAU-XLM” with consistently high levels in “NAU-YZH” and lower but equally significant amounts recorded in “NAU-YH” and “NAU-XLM”.

Among the three developmental stages, the maximum value of anthocyanin content was recorded at 50 DAS. At this stage, the root and the skin were separated into the respective skin and flesh components. Consistent with other stages, “NAU-YZH” accumulated considerable amounts of anthocyanin in both tissues. Anthocyanin was only detected in the leaf of “NAU-YZH” and “NAU-YH” being significantly high in the latter cultivar. Sufficiently high amount of anthocyanin was recorded in the flesh of “NAU-YZH” closely followed by “NAU-XLM” but barely detectable in the “NAU-YH” flesh. In the skin at 50 DAS, significant anthocyanin amounts were detected in “NAU-YZH” and “NAU-YH” (NAU-YZH>NAU-YH) (Figure 2).

### Identification and isolation of anthocyanin biosynthetic genes (ABGs)

To identify the genes associated with anthocyanin biosynthesis, the anthocyanin biosynthetic pathway was analyzed based on the Kyoto Encyclopedia of Genes and Genomes (KEGG), which divided this pathway into three distinct phases: the general phenylpropanoid biosynthesis pathway (ko00940), the flavonoid biosynthesis pathway (ko00941) and later the specific anthocyanin biosynthetic pathway (ko00942). According to de novo assembled transcriptome (SRX707630), using local blast search and sequences functionally annotated by the KEGG pathway analysis, 20 genes encoding enzymes of the anthocyanin biosynthesis pathway were identified. These included three *PAL* (K10775, EC: 4.3.1.24) syntenic genes (14 unigenes), five *4CL* (K01904, EC: 6.2.1.12) syntenic genes (17 unigenes), four *CHS* (K00660, EC:2.3.1.74) syntenic genes (10 unigenes), two *F3*′*H* (K00475, EC:1.14.11.9) syntenic genes (11 unigenes) and one gene each for *C4H, CHI* (K01859, EC:5.5.1.6), *DFR* (K13082, EC: 1.1.1.219 1.1.1.234), *ANS* (K05277, EC: 1.14.11.19), *F3H* (K05280, EC 1.14.13.21) (8, 6, 6, 11 and 11 unigenes, respectively), and 129 unigenes were found to correspond to genes involved in methylation, glucosylation and glycosylation: *UFGT* (K12338, EC: 2.4.1.298), *UGAT* (K12937, EC: 2.3.1.254), *AT* (K12930, EC: 2.4.1.115), *MT* (K12931, EC: 2.3.1.171), *GT1* (K12938, EC: 2.4.1.-) (Table 1).

**Table 1:** Transcriptome-based identification of genes involved in anthocyanin biosynthesis in radish.

**Enzyme**	**Full gene name**	**EC number**	**Number of Unigenes**	**Gene Annotation**	**Unigene ID**
PAL	*Phenylalanine ammonia lyase*	EC:4.3.1.24	14	*PAL1*	CL2858.Contig3_NAU-YH, Unigene13215_NAU-YH, Unigene29856_NAU-YH, Unigene29857_NAU-YH, Unigene3029_NAU-YH
				*PAL2*	CL2858.Contig2_NAU-YH, CL2858.Contig4_NAU-YH, CL2858.Contig5_NAU-YH, Unigene29855_NAU-YH, CL2858.Contig1_NAU-YH
				*PAL4*	Unigene17724_NAU-YH, Unigene17857_NAU-YH, Unigene17723_NAU-YH
C4H	*Cinnamate 4-hydroxylase*	EC:1.14.13.11	8	*C4H*	CL3932.Contig4_NAU-YH, CL3932.Contig5_NAU-YH, Unigene10552_NAU-YH, Unigene11266_NAU-YH, CL3932.Contig1_NAU-YH, CL3932.Contig2_NAU-YH, CL3932.Contig3_NAU-YH, Unigene29684_NAU-YH
4CL	*4-coumarate–CoA*	EC:6.2.1.12	17	*4CL1*	Unigene28587_NAU-YH, Unigene28588_NAU-YH
				*4CL4*	Unigene26090_NAU-YH, Unigene11346_NAU-YH, Unigene1876_NAU-YH
				*4CL3*	Unigene24485_NAU-YH, Unigene24486_NAU-YH, Unigene24487_NAU-YH, Unigene24488_NAU-YH, CL6005.Contig1_NAU-YH, CL6005.Contig2_NAU-YH
				*4CL10*	CL10957.Contig1_NAU-YH, CL2343.Contig1_NAU-YH
				*4CL-LIKE*	CL5789.Contig1_NAU-YH, CL5789.Contig2_NAU-YH, CL11872.Contig1_NAU-YH, CL11872.Contig2_NAU-YH
CHS	*Chalcone synthase*	EC:2.3.1.74	10	*CHS1*	CL14029.Contig2_NAU-YH, Unigene4661_NAU-YH, Unigene9761_NAU-YH, CL14029.Contig1_NAU-YH, CL2470.Contig2_NAU-YH, Unigene5006_NAU-YH
				*CHS3*	CL2470.Contig1_NAU-YH, Unigene37218_NAU-YH
				*CHS8*	Unigene3641_NAU-YH
				*CHS5*	Unigene5900_NAU-YH
CHI	*Chalcone—flavonone isomerase*	EC:5.5.1.6	6	*CHI*	CL12905.Contig1_NAU-YH, CL12905.Contig2_NAU-YH, CL4842.Contig1_NAU-YH, CL4842.Contig2_NAU-YH, Unigene12692_NAU-YH, Unigene14025_NAU-YH
F3H	*Flavanone 3-hydroxylase/Flavonol synthase*	EC:1.14.11.23	11	*F3H*	Unigene17936_NAU-YH, Unigene17937_NAU-YH, CL6897.Contig2_NAU-YH, CL7327.Contig5_NAU-YH, Unigene10168_NAU-YH, Unigene10580_NAU-YH, Unigene11483_NAU-YH, Unigene13117_NAU-YH, Unigene22378_NAU-YH, Unigene34749_NAU-YH, Unigene3501_NAU-YH
F3'H	*Flavonoid 3‘ hydroxylase*	EC:1.14.13.21	11	*F3'H1*	Unigene8907_NAU-YH, Unigene8883_NAU-YH, Unigene8884_NAU-YH, CL1992.Contig1_NAU-YH, CL2046.Contig3_NAU-YH
				*F3'H2*	CL2785.Contig1_NAU-YH, Unigene1076_NAU-YH, Unigene1361_NAU-YH, Unigene210_NAU-YH, Unigene2815_NAU-YH, Unigene8885_NAU-YH
DFR	*Dihydroflavonol 4-reductase*	EC:1.1.1.219 1.1.1.234	6	*DFR*	CL1858.Contig1_NAU-YH, CL1858.Contig2_NAU-YH, CL1858.Contig3_NAU-YH, Unigene20944_NAU-YH, Unigene34696_NAU-YH
				*DFR-LIKE*	Unigene5033_NAU-YH
ANS	*Anthocyanidin synthase/leucoanthocyanidin dioxygenase*	EC:1.14.11.19	11	*ANS*	CL12532.Contig1_NAU-YH, CL12532.Contig2_NAU-YH, CL6245.Contig1_NAU-YH, CL6593.Contig1_NAU-YH, CL6593.Contig2_NAU-YH, CL8001.Contig1_NAU-YH, Unigene13296_NAU-YH, CL13551.Contig1_NAU-YH, CL6549.Contig1_NAU-YH, Unigene4289_NAU-YH, Unigene4558_NAU-YH
ANR	*Anthocyanidin reductace*	EC:1.3.1.77	1	*ANR*	Unigene4158_NAU-YH
UFGT	*UDP-glucosyl transferase*	EC:2.4.1.-	147	*UFGT*	CL1797.Contig1_NAU-YH, CL1797.Contig2_NAU-YH, Unigene3464_NAU-YH, Unigene12158_NAU-YH, Unigene17156_NAU-YH, Unigene11916_NAU-YH, CL8206.Contig1_NAU-YH, CL7501.Contig1_NAU-YH, CL5504.Contig1_NAU-YH
GST	*Glutathione S-transferase*	EC:2.5.1.18	61	*GST*	CL1126.Contig1_NAU-YH, CL1126.Contig2_NAU-YH, CL11539.Contig1_NAU-YH, CL11539.Contig2_NAU-YH, CL12204.Contig1_NAU-YH, CL12204.Contig2_NAU-YH, CL1696.Contig1_NAU-YH, CL1696.Contig2_NAU-YH, CL1696.Contig3_NAU-YH, CL1696.Contig4_NAU-YH, CL1696.Contig5_NAU-YH
OMT	*Flavone 3′-O-methyl transferase*	EC:2.1.1.76	24	*OMT*	CL13021.Contig1_NAU-YH,CL13021.Contig2_NAU-YH, CL2449.Contig1_NAU-YH, CL2449.Contig2_NAU-YH, CL2449.Contig3_NAU-YH, CL2449.Contig4_NAU-YH, CL88.Contig1_NAU-YH, CL88.Contig2_NAU-YH, CL9532.Contig1_NAU-YH, CL9532.Contig2_NAU-YH, CL9933.Contig1_NAU-YH, CL9933.Contig2_NAU-YH, Unigene13005_NAU-YH, Unigene13175_NAU-YH

A total of 102 assembled unigenes were annotated as those that correspond to enzymes involved in the upstream anthocyanin biosynthesis pathway. Specifically, 39 assembled unigenes were found to correspond to the genes in the general phenylpropanoid pathway. From malonyl-CoA to the colored unstable flavonoids, 55 unigene sequences related to six enzymes involved in the flavonoid biosynthetic pathways were isolated. One-twenty nine unigenes were found to be associated with the glycosylation of different anthocyanin aglycons in the specific pathway (Table S4). In the downstream steps of the anthocyanin biosynthesis pathway, stable anthocyanins are trafficked to the anthocyanic vacuole via the non-covalent activity of glutathione S-transferase (GST, EC: 2.5.1.18); 61 unigenes were found to correspond to different *GST*s.

### Cloning and sequence analysis of genes involved in anthocyanin biosynthesis of radish

Partial DNA fragments or full-length sequences of 15 genes encoding anthocyanin biosynthesis-related enzymes were isolated through cloning. Full length genomic fragments of nine genes (*RsCHS3, RsCHI, RsF3H, RsF3*′*H1, RsANS, RsANR, RsUFGT78D2, RsGSTU5*, and *RsGSTU10)*, and partial fragments of six genes (*RsPAL1, Rs4CL3, RsSAM, RsOMT, RsTT12*, and *RsDFR)*, were isolated. The sequences of these genes were submitted to the National Center for Biotechnology Information (NCBI)/GenBank database under the following accession numbers: *RsCHS3* (MF182893), *RsCHI* (MF182892), *RsF3H* (MF182895), *RsF3*′*H1* (MF182896), *RsANS* (MF182899), *RsANR* (MF182891), *RsUFGT78D2* (MF183115), *RsTT12* (MF182901), *RsGSTU5* (MF182897), *RsGSTU17* (MF182898), *RsPAL1* (MF285801), *Rs4CL3* (MF285800), *RsSAM* (MF182902), *RsOMT* (MF182900), and *RsDFR* (MF182894). The length of these gene sequences varied from 900 to 1,579 bp, and the unigene coverage to corresponding genomic sequences was above 93% (Table 2).

**Table 2:** Sequence validation of genes involved in anthocyanin biosynthesis in radish.

**Gene**	**Length**	**Unigenes**	**Coverage (%)**	**Accession number**	**ORF similarity (%)**	**Gap (%)**
*RsPAL1*	1,228	6	97	MF285801	96	3
*Rs4CL3*	1,049	6	99	MF285800	96	1
*RsCHS3*	1,303	2	93	MF182893	98	7
*RsCHI*	1,269	6	100	MF182892	98	0
*RsF3H*	1,356	11	100	MF182895	94	0
*RsF3′H1*	1,579	5	99	MF182896	99	1
*RsDFR*	1,054	5	99	MF182894	100	1
*RsANS*	1,191	11	98	MF182899	99	2
*RsANR*	1,048	1	99	MF182891	100	1
*RsUFGT78D2*	1,240	2	100	MF183115	100	0
*RsOMT*	1,102	1	100	MF182900	100	0
*RsSAM*	1,170	2	100	MF182902	89	0
*RsTT12*	1,146	3	100	MF182901	98	0
*RsGSTU5*	900	1	99	MF182897	99	1
*RsGSTF17*	1,223	2	99	MF182898	99	1

Genomic sequence analysis depicted the different domains of each of the ABGs, which bear the catalytic sites that enable the unique reactions performed by each gene. Intron classification revealed that most of the anthocyanin biosynthetic genes bear phase one introns with predominantly asymmetrical exons, except *RsSAM* which has one symmetrical (1-1) exon (Figure S2). The analysis also revealed unique features in the flavanone hydroxylase genes; the *RsF3*′*H1* gene located on scaffold 45 is intronless, while *RsF3H* bears exclusively phase 0 introns. The *RsSAM* ABG has the highest number of introns (10) and the isoelectric points of radish ABGs are between 4.589 (*RsCHI*) and 11.87 (*RsPAL1*), which categorizes the radish ABGs as stable (Table 3).

**Table 3:** Characteristics of anthocyanin biosynthesis genes in radish.

**Gene**	**Scaffold**	**Strand**	**Start-end**	**CDS length^a^**	**Number of exons^b^**	**Protein size**	**Theoretical MW(kDa)**	**Theoretical PI**
*RsPAL1*	Rs_scaf92	(+)	243886-247511	2,400	3	756	85,897.42	11.874
*RsPAL2*	Rs_scaf801	(−)	53717-56216	2,175	1	724	78,601.91	6.194
*RsPAL4*	Rs_scaf132	(−)	77225-80788	2,125	2	702	76,795.77	5.865
*Rs4CL1*	Rs_scaf707	(−)	80817-83472	1,659	3	552	60,216.51	5.263
*Rs4CL4*	Rs_scaf34	(−)	351971-356525	1,782	5	593	65,050.21	5.039
*Rs4CL3*	Rs_scaf424	(−)	13415-20270	1,677	10	558	60,552.82	6.117
*Rs4CL10*	Rs_scaf4963	(−)	3583-5666	1,545	4	514	55,334.04	6.183
*RsCHS1*	Rs_scaf14	(+)	335667-337099	1,191	1	396	43,052.88	6.671
*RsCHS3*	Rs_scaf11	(−)	1269822-1271094	1,188	1	395	42,982.62	6.145
*RsCHS5*	Rs_scaf119	(−)	138269-139725	1,991	1	396	43,094.76	6.277
*RsCHS8*	Rs_scaf217	(−)	277834-279184	1,101	1	366	39,757.1	5.948
*RsCHI*	Rs_scaf7714	(−)	522-2902	618	3	206	26,620.36	4.589
*RsF3H*	Rs_scaf58	(+)	29065-30370	1,077	2	358	40,087.57	5.465
*RsF3′H1*	Rs_scaf45	(+)	58827-60401	1,575	0	524	59,512.06	6.622
*RsF3′H2*	Rs_scaf62	(−)	524013-524884	871	2	156	37,572.63	10.858
*RsDFR*	Rs_scaf2809	(−)	21463-23363	924	5	306	33,883.97	5.627
*RsANS*	Rs_scaf190	(−)	249786-250944	1,074	1	357	40,810.16	5.747
*RsUFGT*	Rs_scaf749	(−)	40726-42222	1,383	1	460	50,932.69	4.983
*RsANR*	Rs_scaf197	(−)	78407-79919	1,017	5	338	37,799.2	5.016
*RsGSTF11*	Rs_scaf1076	(−)	945-2916	987	4	328	37,159.2	6.273
*RsGSTU10*	Rs_scaf648	(+)	14594-15478	642	1	213	24,185.78	7.041
*RsGSTU5*	Rs_scaf10	(−)	594776-595590	621	1	207	24,273.96	7.685
*RsOMT*	Rs_scaf464	(−)	133367-135867	1,495	3	364	39,906.35	5.76
*RsSAM*	Rs_scaf 831	(+)	88381-91197	930	10	309	34,158	6.373

a *The CDS length was based on predicted sequences*.

b *The number of exons was based on the genomic data*.

### Transcript profiling of ABGs in four radish genotypes

**Figure 3:**
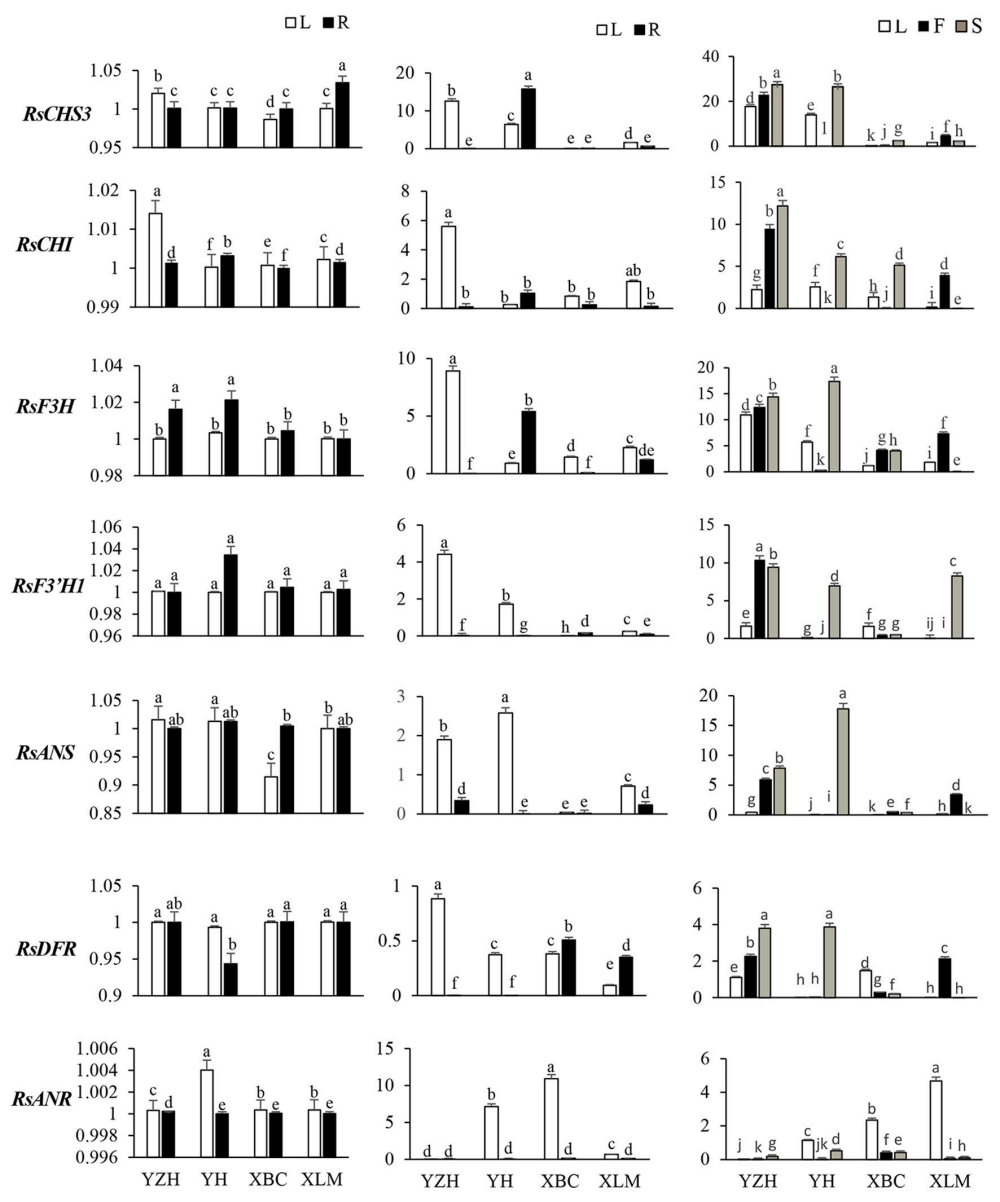
Spatial temporal transcriptional analysis of genes associated with anthocyanin biosynthesis in four cultivars of *R. sativus* using qRT-PCR. Four tissues were used: the leaf (L), young root (R), flesh (F) and skin (S). Relative gene expression levels were normalized against *actin* transcript levels. Values connected by the same letter within the same gene and data collection point are not significantly different at *P* ≤ 0.05. The transcriptional levels of the same gene in the leaf and root tissues at 10, 30, and 50 DAS are expressed on the y axes while radish genotypes are expressed on the x axis.

RT-qPCR was utilized to analyze transcript levels of 14 radish genes associated with anthocyanin biosynthesis, relative to the cultivar, developmental stage and plant tissue. Four color variant radish genotypes (“NAU-YZH”, “NAU-YH”, “NAU-XBC” and “NAU-XLM”) at three developmental stages (10, 30, and 50 DAS) and the leaf, young root, taproot flesh and skin were used. The initial steps of the flavonoid pathway from 4-coumaroyl CoA through chalcone and naringenin to dihydroflavonol are catalyzed by CHS, CHI, F3H, and F3′H (Tanaka et al., 2008). *RsCHS3* and *RsCHI* abundantly expressed in the leaf and root tissues of all genotypes under study, with the highest expression in “NAU-YZH” leaf and “NAU-XLM” root at 10 DAS (Figure 3). The latter gene was 12-folds up regulated at 30 DAS characterized by high levels in “NAU-YZH” leaf and “NAU-YH” root, and non-traceable in all tissues of “NAU-XBC” while there was a general dramatic decline of *RsCHI* in the root (Figure 3). At 50 DAS, however, the expression levels of *RsCHS3* increased by 2-folds with consistent high expression in “NAU-YZH” and “NAU-YH” leaf, flesh and skin, respectively (Figure 3). *RsCHI* on the other hand generally showed no change in transcript levels with significant high expression in the leaf of “NAU-YH” and the skin and root of “NAU-YZH”. Notably, while the lowest transcripts of *RsCHI* significantly were expressed in “NAU-XLM” leaf, this gene was increased by 4-folds in the flesh at 50 DAS (Figure 3).

The transcripts of *RsF3H* were equally expressed in both leaf and root but with significantly higher expression of *RsF3*′*H1* in “NAU-YZH” and “Nau-YH” at 10 DAS (Figure 3). However, *RsF3*′*H* peaked in the leaf and was barely detected in the root at 30 DAS, while *RsF3H* remained consistently up regulated with high expression in “NAU-YZH” leaf and “NAU-YH” root (Figure 3). There was no change in transcript levels of *RsF3H* but *RsF3*′*H1* globally increased by 10-folds, and both genes showed a consistent high expression in the leaf and skin of “NAU-YZH” and “NAU-YH” respectively, at 50 DAS (Figure 3). Transcripts of *RsF3H* were notably significantly repressed in the leaf and skin but increased by 5-folds in the flesh of “NAU-XLM”.

In the synthesis of dihydroflavanols, which are intermediates in anthocyanin biosynthesis, the genes *RsDFR* and *RsANS* play critical synthesis functions, while *RsANR* acts as an inhibitory gene to *RsANS*. The *RsANS* gene was down-regulated in the leaf of “NAU-XBC”, and up-regulated in “NAU-YZH” and “NAU-YH” with no significant variation in the root tissues at 10 DAS (Figure 3). Transcript levels increased in the leaves, against traceable levels in the root at 30 DAS and continued to increase at 50 DAS while remaining highly expressed in “NAU-YZH” leaf, flesh and skin, “NAU-XLM” flesh and “NAU-YH” skin (Figure 3). In all genotypes under study, transcript levels of *RsDFR* were not variable in the 10 DAS and 30 DAS leaves and 10 DAS root but with the exception of “NAU-XBC”, decreased in the root at 30 DAS (Figure 3). However, *RsDFR* expression steadily increased at 50 DAS with the highest levels depicted in the “NAU-YZH” and “NAU-XLM” flesh and “NAU-YZH” and “NAU-YH” skin (Figure 3). There was a high expression of *RsANR* in “NAU-YH” leaf and over 10-fold expression in “NAU-XBC” leaves. Over 6-fold increased expression was recorded in the leaf of “NAU-XLM” but silenced in the root tissues at 50 DAS (Figure 3).

**Figure 4:**
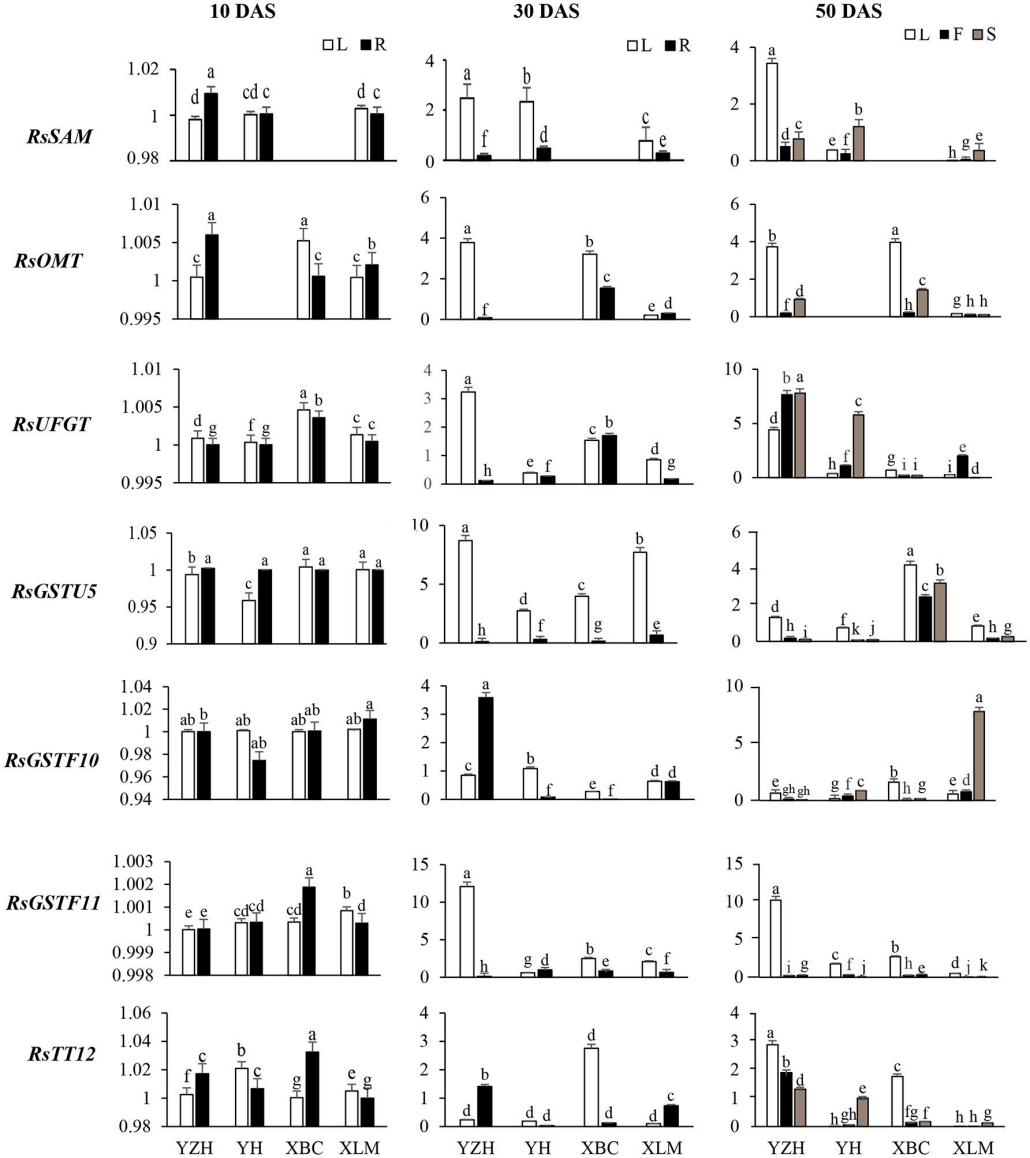
Spatial temporal transcriptional analysis of genes associated with anthocyanin biosynthesis in four cultivars of *R. sativus* using qRT-PCR. Four tissues were used: the leaf (L), young root (R), flesh (F) and skin (S). Relative gene expression levels were normalized against *actin* transcript levels. Values connected by the same letter within the same gene and data collection point are not significantly different at *P* ≤ 0.05. The transcriptional levels of the same gene in the leaf and root tissues at 10, 30, and 50 DAS are expressed on the y axes while radish genotypes are expressed on the x axis.

*RsSAM, RsOMT*, and *RsUFGT* are involved in glucosylation and methylation of anthocyanidins, resulting in stable compounds. Transcripts of *RsSAM* were highly expressed in tissues of “NAU-YZH” and the colored tissues of “NAU-YH” and “NAU-XLM”, and undetectable in all tissues of “NAU-XBC” at 30 and 50 DAS (Figure 4). *RsOMT* on the other hand was significantly expressed in the “NAU-YZH” and “NAU-XLM” with higher expression in the root and the leaf at 10 and 30 DAS, respectively. Notably, transcripts of *RsOMT* were significantly elevated at 50 DAS in the white colored “NAU-XBC” (Figure 4). Nevertheless, *RsUFGT* consistently increased across the three developmental stages, with consistent high expression in the colored radish cultivars, although the expression dramatically decreased in the root of “NAU-YH” at 30 DAS (Figure 4). Generally, the transcripts of *RsSAM, RsOMT*, and *RsUFGT* were upgraded in tissues expressing high anthocyanin content.

There was no distinct variation in the glutathione transferase transcripts at 10 DAS, but their levels heightened at 30 DAS in the leaf and were barely detectable in the root at this stage (Figure 4). However, *RsGSTF11* was over 15-folds up-regulated in the leaf, with a constant up-regulation in all tissues of the “NAU-YZH” at 50 DAS. On the other hand, *RsGSTU5* and *RsGSTF10* transcript levels increased in the skin with an up-regulation in the “NAU-XBC” and “NAU-YH” flesh and skin, respectively (Figure 4).

### Correlation between gene expression and total anthocyanin

**Figure 5:**
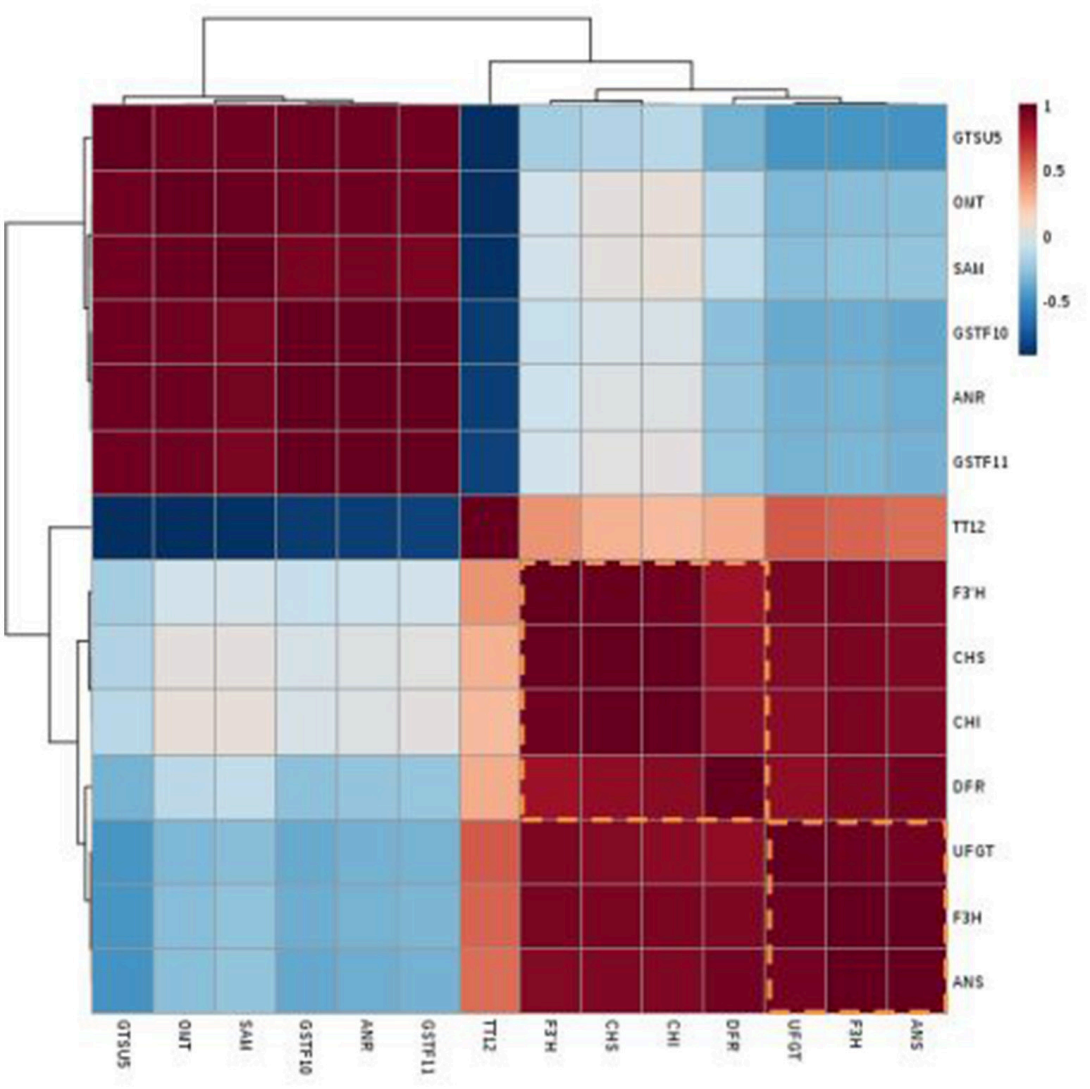
Heat map of the clustered correlations between anthocyanin accumulation patterns and gene expressions in red radish. Three sampling stages, three biological replicates, skin and flesh tissues were treated as independent factors in anthocyanin-transcript pairwise comparisons, which were carried out for 21 transcriptional data points for each gene.

The genotype “NAU-YZH” was used to analyze the relationship between transcript levels and total anthocyanin content. Three sampling points, three biological replicates and the leaf, young root (10 and 30 DAS), leaf, taproot skin and flesh tissues (50 DAS) were regarded as independent factors in the transcript pairwise comparisons of total anthocyanin, yielding 21 transcriptional data points for each gene (Figure 5). Pearson's correlation coefficient was used for the correlation analysis.

Transcripts of *RsF3H, RsUFGT, RsANS*, and *RsF3*′*H1*, showed a significant correlation (*r* > 0.85) with total anthocyanin content (Table S3).The co-expression patterns between the gene expression and anthocyanin contents were generated and are depicted in a heat map of clustered correlations (Figure 5). The set of genes from number 8–11 had the strongest positive correlation with total anthocyanin, including *RsF3H* (*r* = 0.92), *RsUFGT* (*r* = 0.92), *RsF3*′*H1* (*r* = 0.84), and *RsANS* (*r* = 0.89) while those in lines 1–4 exhibited the strongest negative correlation: *RsGSTU5* (*r* = 0.62), *RsSAM* (*r* = 0.5), *RsGSTF10* (*r* = −0.47), and *RsANR* (*r* = −0.47) (Table S3). Linear regressions performed between the cumulative transcription of each of the 14 genes and the corresponding anthocyanin contents were shown in Figure S3. These data indicated that *RsF3H, RsUFGT, RsANS*, and *RsF3*′*H* might be candidate genes ascribed to the red pigment of the “NAU-YZH” root.

## Discussion

### Total anthocyanin content in tissues of four radish genotypes

The anthocyanin concentrations of radish under study increased with increase in days after sowing, resulting in higher concentrations in mature taproots than sprouts of colored tissues. There were variations in the levels of anthocyanin in the skin and flesh organs of the four different radish cultivars. The increase in anthocyanin accumulation as the crop advances in growth could be due to increased accumulation of anthocyanin content in tissues, resulting from increase in size and hence the capacity of the plant's biosynthetic machinery. It is speculated that this occurrence is associated with the coordination of plant metabolism because of the ability of plants to regulate the metabolism in respective organs at various developmental stages (Majdi et al., 2014). These results concur with those found in yam, cherry, grape, and chili (Aza-Gonzalez et al., 2013; Liu et al., 2013; Xie et al., 2015; Yin et al., 2015). There was also a difference in the accumulation of anthocyanin in tissues of different radish at each developmental stage. For instance in “NAU-YH” anthocyanin in the skin was higher than in the flesh, while “NAU-XLM” recorded higher anthocyanin content in the flesh and significantly low amounts in its skin, but these contents increased progressively with growth, though at varying rates.

### Characterization and expression of ABGs in radish

The length of cloned gene sequences from our study varied from 900 to 1,579 bp, and the unigene coverage to corresponding genomic sequences was above 93%, indicating that the radish anthocyanin unigenes from RNA-seq were successfully assembled and were viable for further investigation.

Gene structure analysis predicted the significant domains in each gene, which help to explain their mechanism of action. For instance, the distinct substrate specificity of the NAD_binding _4 domain of *RsDFR* with ASN-active catalytic site explains the preferential accumulation of pelargonidin and cyanidin derivatives instead of malvidin or petunidin (Shimada et al., 2005). The analysis also revealed that *RsF3*′*H* is intronless and that most of the ABGs bear phase one introns with predominantly asymmetrical exons, except *RsSAM* which has one symmetrical (1-1) exon. Transcript abundance of *RsF3*′*H* was found to be consistently lower when compared to *RsF3H*. Introns have been found to contribute to increased protein abundance while elimination of introns renders protein product undetectable. This impeccable intronic function is referred to as intron-mediated enhancement (Akua et al., 2010). Extentive analyses further confirmed these inferences where genes bearing introns in yeast were found to have enhanced mRNA and protein levels than intronless genes (Juneau et al., 2006). The pattern of spliceosomal introns has a significant relationship with the conservation of splice signals sequence of exons, therefore, the relatively dominated phase 1 introns in the ABGs in radish indicates a relative reduction in conservation of these genes (Long and Deutsch, 1999).

Despite the consistent occurrence of all ABGs in any studied variety, a species-specific control of the genes of the basic pathway and at key branching points is assumed to contribute to the differences in anthocyanin content and the shift from lighter to darker hues as the crop develops (Ageorges et al., 2006). In the present study, variations in anthocyanin coloration across tissues of four radish genotypes are ascribable to alterations in the unique expression patterns of the overall set of anthocyanin genes.

The transcripts of ABGs portrayed a commensurate expression for most of the genes in the leaf and root tissues of the four radish cultivars at 10 DAS, revealing that ABGs are coordinately expressed at early stages of development. However, the expression of anthocyanin pathway genes is consistent with the accumulation of anthocyanins: as the plant begins to pigment, the expression of key anthocyanin biosynthesis genes is detected to obviously increase.

### Expression of ABGs involved in primary flavonoid upstream pathway

*Chalcone synthase* (*CHS)* and *Chalcone isomerase* (*CHI)* are the first genes in the flavonoid branch of the anthocyanin biosynthesis pathway. *RsCHS3* recorded the highest level of transcripts when compared to all the other genes and consistently increased throughout plant development. Its transcript abundance correlated positively with total anthocyanin, signifying that it is a key gene for anthocyanin synthesis, findings that echo previous research (Park et al., 2011; Chen et al., 2016). The loss of the *RsCHS3* gene from the 30 DAS stage, through 50 DAS, in the “NAU-XBC” could perhaps be the single most factor contributing to the loss of color in this cultivar. A knockout mutation of the gene impeded anthocyanin accumulation in seeds resulting in a transparent testa in *Arabidopsis* (Shirley et al., 1995). Although *RsCHI* correlated positively with anthocyanin content, its transcription levels were markedly down- regulated in the root at 50 DAS, being non-consistent with previous reports (Xu et al., 2014). This result indicates that a single gene is not responsible for anthocyanin accumulation and that anthocyanin biosynthesis involves the coordinated mechanism of many genes (Walker et al., 2007; Yu et al., 2012).

The transcription of *RsF3*′*H1* seems to have been developmentally activated after pre-cortex splitting (30 DAS) in “NAU-YZH”, “NAU-YH” skin, and “NAU-XLM” flesh, but with lower transcript levels than *RsF3H* and was barely transcribed in “NAU-XBC”, which is white colored. However, the expression of *RsF3H* and *RsF3*′*H1* greatly increased in all cultivars at maturity except for the non-pigmented, taproot skin of “NAU-XLM”, taproot flesh of “NAU-YH” and tissues of “NAU-XBC”. In the latter cultivar, the levels of transcripts remained low but detectable. Striking variations were also observed in the regulation of the flavonoid hydroxylase genes. The expression profile of *RsF3H* was relatively high even before 50 DAS and transcripts of this gene were present at maturity in all cultivars, including the white cultivar “NAU-XBC”.

The reciprocal expression levels of these flavonoid hydroxylase genes most likely contribute to different color hues in radish. Previous studies also proposed a plausible cause of color transition from pelargonidin type in “NAU-YZH” to cyanidin type in “NAU-XLM” which could be due to a reduced *RsF3*′*H1* mRNA level (Mudalige-Jayawickrama et al., 2005) resulting in the inefficient production of dihydroquercitin and increased dihydrokaempferol and subsequently, pink-purple coloration. Differential expression of transcripts encoding flavonoid-hydroxylase was also reported in mulberry (*Morus alba L*), cauliflower (*Brassica oleraceae* var *botrytis*), and Chinese bayberry (*Myrica rubra*) (Chiu et al., 2010; Niu et al., 2010; Li et al., 2014). Notably, *RsF3H* and *RsCHI* were clearly expressed in the white-flesh radish “NAU-XBC”, but anthocyanin accumulation was not detected. These findings suggest that *RsF3H* and *RsCHI* are likely more highly regulated than the other anthocyanin biosynthesis structural genes. Additionally, findings from other studies indicate that *CHI* and *F3H* are early genes in the anthocyanin biosynthetic pathway and are coordinately expressed, with increased transcript levels toward plant maturity (Ravaglia et al., 2013).

### Expression of ABGs involved in anthocyanin modification specific pathway

Dihydroflavonol 4-Reductase (DFR) encoded by a single gene (*DFR*) converts dihydroflavonols to leucoanthocyanidins. Besides the high expression in colored tissues, this gene was also found to be well expressed in the white tissues of “NAU-XBC”, and is, therefore, likely to participate in the synthesis of other secondary metabolites like free-auxins (Shen et al., 2013). These results relate to those obtained in litchi and kiwifruit (Montefiori et al., 2011; Wei et al., 2011).

Anthocyanidin synthase/leucoanthocyanidin oxidase (LDOX) catalyzes the conversion reaction of leucoanthocyanidins to colored anthocyanidins. However in this study, in spite of the high expression of *RsANS* in the early developing stage, the leaf color of three colored radishes is green. This could result from the catalysis of Anthocyanidin synthase (ANS) substrate into flavonol or/and the inhibitory role of the *RsANR*, which converts the colored anthocyanidins to epicatechin, resulting in redirection of anthocyanin pathway into proanthocyanidin pathway (Jaakola et al., 2002). Our study is consistent with findings from bilberry (Jaakola, 2013). However in the later stages *ANS* is highly expressed in colored tissues and at stages coinciding with elevated anthocyanin amounts, consistent with previous findings (Zhang et al., 2015).

Down-stream genes such as glucosyltransferases greatly influence the direction of anthocyanin synthesis through the regulation of anthocyanidin glucosyltransferase in thickening radish taproot.

It was found that the transcription level of *RsUFGT* was much lower in the white-flesh cultivar “NAU-XBC” than in the red and pink colored radishes. Similar to *RsCHS3*, our results also showed that the mRNA levels encoding *RsUFGT*, the specific gene for anthocyanin biosynthesis, increased proportionally to the anthocyanin content across all the three developmental stages, suggesting that these two genes are under a different regulatory regime, in comparison to the other ABGs in radish, and that the biosynthesis of anthocyanin is controlled at an earlier stage as reported in previous studies (Kobayashi et al., 2001). The expression of O-Methyltransferase (*RsOMT)* in the red colored “NAU-YZH” was higher than that of *RsUFGT*, which was also well expressed in all genotypes, unlike *RsOMT*. The purple-pinkish colored “NAU-XLM” expressed comparatively lower levels of *OMT* when compared to *RsUFGT*. It has been proposed that the methylation of B-ring hydroxyl groups causes a shift toward deeper red colors (Tanaka et al., 2008). Therefore, it can be inferred that the relative abundance of *RsF3H* to *RsF3*′*H1* and *RsOMT* to *RsUFGT* could *per se* explain to a greater extent the phenotypic variation of anthocyanin content, color hue and color intensity in radish. Secondly, the key regulation point for quantitative anthocyanin variation is in the downstream pathway at the *RsUFGT* level, but the qualitative differences are precisely controlled upstream of *RsUFGT* at the flavonoid hydroxylases' level and at *RsOMT* which is downstream of *RsUFGT*.

### ABGs involved in transportation and localization of anthocyanins

The sequestration of anthocyanin from the cytoplasm to the vacuole is poorly understood, and various mechanisms have been put across to explain the process, including transport proteins like GSTs and MATE transporters. The GST*s* encoded by a group of *Glutathione-S-transferase* genes, whose specific functions remain to be elucidated in radish, have been implicated in the transport of anthocyanin in other crops (He et al., 2010; Gomez et al., 2011). In this study, the featured GSTs were found to positively coincide with total anthocyanin and exhibit similar expression patterns to the *RsUFGT*, although the correlation between cumulative transcription and total anthocyanin throughout crop development was lower. The abundance of GST transcripts in the present study exhibited genotypic specificity, as *RsGSTF10* was found to correlate with elevated anthocyanin in “NAU-XLM”, *RsGSTU5* with “NAU-YZH” and *RsGSTF11* with “NAU-YZH”, “NAU-YH” and “NAU-XLM”. *RsGSTU5* may contribute to spatial differential accumulation in red radish, owing to its elevated transcript levels in the root against the suppressed transcript levels of key ABGs at 30 DAS. In anthocyanin transport, it has been suggested through mutant analysis that the anthocyanin defective mutants were unable to accumulate anthocyanins into the vacuoles (Conn et al., 2008) implying that glutathione transferases are possible anthocyanin transporters.

At 30 DAS, the red colored radish accumulated significant amounts of anthocyanin in the root despite the down regulation of major ABGs in this tissue. It was also found that *TT12*, a MATE transporter was consistently up-regulated in the red colored radish but low amounts in the white colored tissues. *TT12* was reported to mediate anthocyanin transportation in *Arabidopsis* (Marinova et al., 2007).

To our knowledge, this is the first report describing the spatial-temporal expression patterns of anthocyanin biosynthetic genes (ABGs) in radish. Our results demonstrate the coordinated expression of ABGs in relation to anthocyanin accumulation in radish tissues and that there may be a common regulatory mechanism governing the coordinated expression of related genes. Furthermore, it appears that the major control point to anthocyanin biosynthesis in radish is *UFGT*. Globally, the correlation of anthocyanin content with coordinated gene regulation would be the key contributing factor to phenotypic and spatial-temporal anthocyanin accumulation in radish.

## Author contributions

MM and LL designed the experiments and wrote the manuscript. MM, LF, YC and WZ performed validation experiments. YW contributed powerful analytical tools. XZ and KK contributed to proofreading of this manuscript. XL and LL conceived the study and managed the experiments. All authors read and approved the final manuscript.

### Conflict of interest statement

The authors declare that the research was conducted in the absence of any commercial or financial relationships that could be construed as a potential conflict of interest.

## Supplementary material

Figure S1 The proposed anthocyanin biosynthesis pathway. Anthocyanins are synthesized by a multienzyme complex loosely associated to the endoplasmic reticulum in the plant cytosol (PAL, phenylalanine ammonia lyase; C4H, cinnamate 4-hydroxylase; CHS, chalcone synthase; CHI, chalcone isomerase; F3H, flavanone 3-hydroxylase; F3′H, flavonoid 3′-hydroxylase; DFR, dihydroflavonol reductase; ANS, anthocyanidin synthase,; UFGT, UDP-glucose flavonoid 3-*O*-glucosyltransferase; MT, methyltransferase; GST, glutathione s- transferase). Proanthocyanidins (PAs) are synthesized when the pathway branches off the anthocyanin pathway, catalyzed by enzymes such as ANR, anthocyanidin reductase. The numbers in the brackets are unigenes corresponding to the genes in the radish transcriptome.

Figure S2 Gene structure and predicted functional domains of radish anthocyanin biosynthetic genes. Functional domains were predicted in SMART (http://smart.embl-heidelberg.de/smart/set_mode.cgi?) NORMAL = 1. Gene structures were displayed by Gene Structure Display Server (http://gsds.cbi.pku.edu.cn/).

Figure S3 Linear regression between the cumulative gene transcription and total anthocyanin content in “NAU-YZH”.

Table S1 Gene specific primers used for cloning.

Table S2 Primers used for RT-qPCR.

Table S3 The r and p values of the correlations between anthocyanin accumulation patterns and gene expression in “NAU-YZH”.

Table S4 List of unigenes involved in anthocyanin biosynthesis pathway of radish in the transcriptome database.

## Abbreviations

4CL: Courmarate 4-ligase
ANR: Anthocyanidin reductase
ANS: Anthocyanidin synthase
CHI: Chalcone isomerase
CHS: Chalcone synthase
DAS: Days after sowing
DFR: Dihydroflavonol reductase
F3′H: Flavonoid 3′-hydroxylase
F3H: Flavanone 3-hydroxylase
GST: Glutathione-S-transferase
OMT: Methyl O-transferase
PAL: Phenylalanine ammonia lyase
SAM: S-Adenosylmethyl Transferase
TT12: Transparent Testa12
UFGT: UDP glucose:flavonoid 3-O-glucosyltransferase.
