# Supplementary figures and images for: Coordinated Regulation of Anthocyanin Biosynthesis Genes Confers Varied Phenotypic and Spatial-Temporal Anthocyanin Accumulation in Radish (Raphanus sativus L.)
Source: Front Plant Sci. 2017 Jul 19;8:1243. doi: 10.3389/fpls.2017.01243 (PMC5515825; doi:10.3389/fpls.2017.01243)

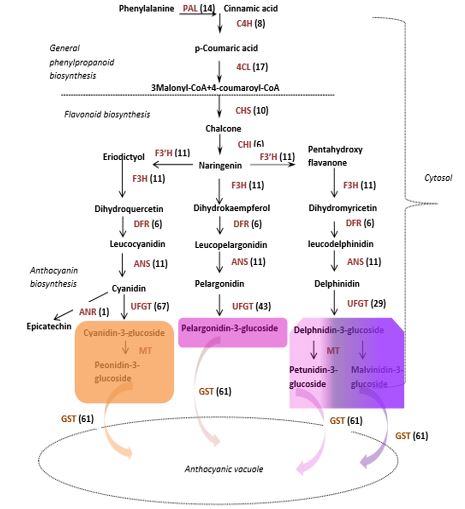

Supplement: Figure S1 — The proposed anthocyanin biosynthesis pathway. Anthocyanins are synthesized by a multienzyme complex loosely associated to the endoplasmic reticulum in the plant cytosol (PAL, phenylalanine ammonia lyase; C4H, cinnamate 4-hydroxylase; CHS, chalcone synthase; CHI, chalcone isomerase; F3H, flavanone 3-hydroxylase; F3′H, flavonoid 3′-hydroxylase; DFR, dihydroflavonol reductase; ANS, anthocyanidin synthase,; UFGT, UDP-glucose flavonoid 3-O-glucosyltransferase; MT, methyltransferase; GST, glutathione s- transferase). Proanthocyanidins (PAs) are synthesized when the pathway branches off the anthocyanin pathway, catalyzed by enzymes such as ANR, anthocyanidin reductase. The numbers in the brackets are unigenes corresponding to the genes in the radish transcriptome. [file Image1.JPEG]

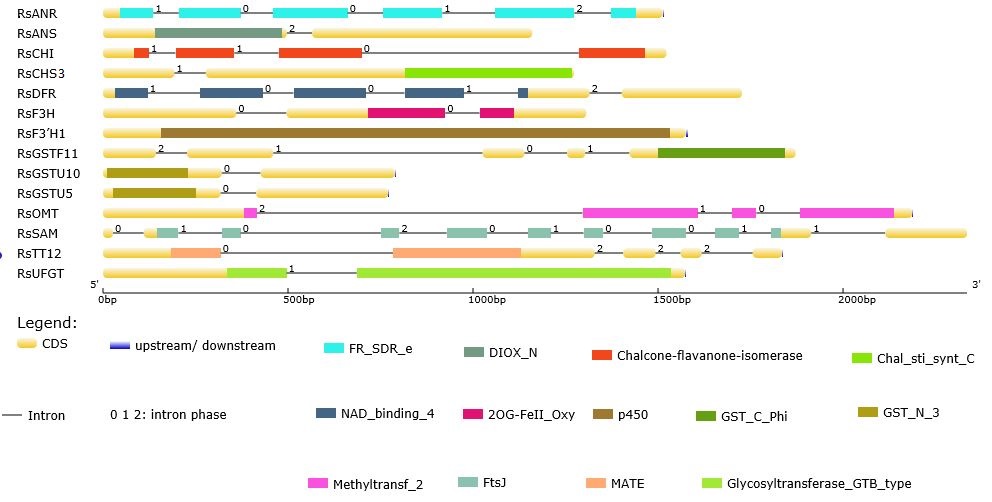

Supplement: Figure S2 — Gene structure and predicted functional domains of radish anthocyanin biosynthetic genes. Functional domains were predicted in SMART (http://smart.embl-heidelberg.de/smart/set_mode.cgi?) NORMAL = 1. Gene structures were displayed by Gene Structure Display Server (http://gsds.cbi.pku.edu.cn/). [file Image2.JPEG]

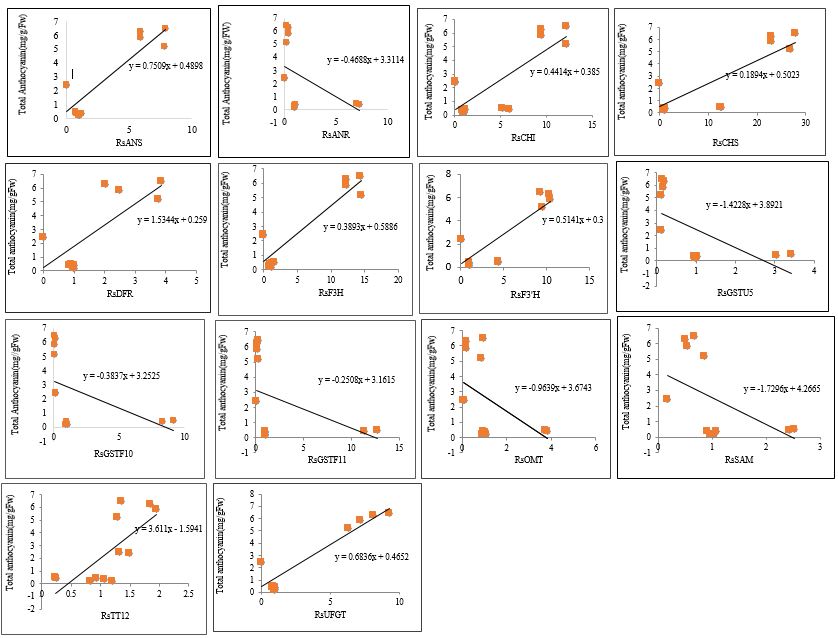

Supplement: Figure S3 — Linear regression between the cumulative gene transcription and total anthocyanin content in “NAU-YZH”. [file Image3.JPEG]
