# Supplementary material for: Coordinated Regulation of Anthocyanin Biosynthesis Genes Confers Varied Phenotypic and Spatial-Temporal Anthocyanin Accumulation in Radish (Raphanus sativus L.)
Source: Front Plant Sci. 2017 Jul 19;8:1243. doi: 10.3389/fpls.2017.01243 (PMC5515825; doi:10.3389/fpls.2017.01243)
Supplement: Table S1 — Gene specific primers used for cloning. [file Table1.DOCX]

**TABLE S1 Gene specific primers used for cloning**

| **Gene name** | **Forward primer (5′-3′)** | **Reverse primer （5′-3′）** |  |
| --- | --- | --- | --- |
| *RsCHS3* | GCCACGCATCGATCAAACTC | AAGGAAGGTAGGTAGGCGCA | |
| *RsANR* | TCTCAAGATCTTAACCTCTCATAGC | ATAGGAATCATTTTACAGCACAGGA | |
| *RsF3'H1* | TTTCACAGGAAGAGGTTGGAACACT | GGACTTGCATTACACAAACATCACA | |
| *RsPAL1* | CCAAACTCCTAAAACGAACA | AGGATCTCGAAACGGATG |  |
| *RsUFGT* | CAAGAAGATGGTTGCAGTTGAAAG | TTCACATGCTGTATAATGACTCAAA | |
| *Rs4CL3* | CTAGTATTATTTTGGTTTGAGTTGG | TCCATTTTTTCCTGTTTCCCCCTTG | |
| *RsSAM* | TACAAAGACATGGGAAAAGC | GGTAAGCCATAGAAACAAACAC | |
| *RsOMT* | CTCACCCATCACACACTCCTC | CTGCTAAATAACCGCAAGACTGT | |
| *RsCHI* | CAACTCAAACCATGTCTTCTT | CCTGGCCAAAGAAAACTGATCCTG | |
| *RsDFR* | CGTGCTTTGCTGGTTGGT | CTCGGGTATAATTGTTCTGT | |
| *RSGSTU5* | ACAACAACACATTTCTAAGAGACGG | TGACTTTCGACTAAGCGGATCTGAT | |
| *RsGSTU17* | ATTCCCTACGATCTACCACTTAAGC | TACACAAGGACCACAACCATCAACT | |
| *RsTT12* | GGAAAATAAGAAAGGGGATC | AGCAAGGGAAAGAGGTCA |  |
| *RsANS* | CAAGAAGATGGTTGCAGTTGAAAG | TTCACATGCTGTATAATGACTCAAA | |
| *RsF3H* | TAAATGGCTCCAGGAACTCTA | AGGAGTCTAAGCGATGATTTG | |
