# Supplementary material for: Coordinated Regulation of Anthocyanin Biosynthesis Genes Confers Varied Phenotypic and Spatial-Temporal Anthocyanin Accumulation in Radish (Raphanus sativus L.)
Source: Front Plant Sci. 2017 Jul 19;8:1243. doi: 10.3389/fpls.2017.01243 (PMC5515825; doi:10.3389/fpls.2017.01243)
Supplement: Table S2 — Primers used for RT-qPCR. [file Table2.DOCX]

**TABLE S 2 Primers used for RT-qPCR**

|  | **Gene name** | **Forward primer (5′-3′)** | **Reverse primer （5′-3′）** |  |
| --- | --- | --- | --- | --- |
|  | *RsCHS* | GCCACGCATCGATCAAACTC | AAGGAAGGTAGGTAGGCGCA |  |
|  | *RsANR* | TCTCAAGATCTTAACCTCTCATAGC | ATAGGAATCATTTTACAGCACAGGA | |
|  | *RsF3'H* | TTTCACAGGAAGAGGTTGGAACACT | GGACTTGCATTACACAAACATCACA | |
|  | *RsGSTF10* | CCAAACTCCTAAAACGAACA | AGGATCTCGAAACGGATG |  |
|  | *RsUFGT* | CAAGAAGATGGTTGCAGTTGAAAG | TTCACATGCTGTATAATGACTCAAA | |
|  | *RsGSTF11* | CTAGTATTATTTTGGTTTGAGTTGG | TCCATTTTTTCCTGTTTCCCCCTTG | |
|  | *RsSAM* | TACAAAGACATGGGAAAAGC | GGTAAGCCATAGAAACAAACAC | |
|  | *RsOMT* | CTCACCCATCACACACTCCTC | CTGCTAAATAACCGCAAGACTGT | |
|  | *RsCHI* | AGGGGACATAACTAATGCTCGGACT | CAAACCAACAATAAGCGTGTTTCAG | |
|  | *RsDFR* | CGTGCTTTGCTGGTTGGT | CTCGGGTATAATTGTTCTGT |  |
|  | *RsGSTU5* | ACAACAACACATTTCTAAGAGACGG | TGACTTTCGACTAAGCGGATCTGAT | |
|  | *RsTT12* | GGAAAATAAGAAAGGGGATC | AGCAAGGGAAAGAGGTCA |  |
|  | *RsANS* | CAAGAAGATGGTTGCAGTTGAAAG | TTCACATGCTGTATAATGACTCAAA | |
|  | *RsF3H* | TAAATGGCTCCAGGAACTCTA | AGGAGTCTAAGCGATGATTTG |  |
