# Supplementary material for: Coordinated Regulation of Anthocyanin Biosynthesis Genes Confers Varied Phenotypic and Spatial-Temporal Anthocyanin Accumulation in Radish (Raphanus sativus L.)
Source: Front Plant Sci. 2017 Jul 19;8:1243. doi: 10.3389/fpls.2017.01243 (PMC5515825; doi:10.3389/fpls.2017.01243)
Supplement: Table S3 — The r and p values of the correlations between anthocyanin accumulation patterns and gene expression in “NAU-YZH”. [file Table3.docx]

**TABLE S3. The r and p values of the correlations between anthocyanin accumulation patterns and gene expression in Nau-YZH.**

|  | *RsF3H* | *RsANR* | *RsUFGT* | *RsCHS3* | *RsGSTF10* | *RsTT12* | *RsDFR* | *RsANS* | *RsCHI* | *RsF3'H1* | *RsOMT* | *RsGTSU5* | *RsGSTF11* | *RsSAM* |
| --- | --- | --- | --- | --- | --- | --- | --- | --- | --- | --- | --- | --- | --- | --- |
| r value | 0.919475 | -0.46514 | 0.918453 | 0.831412 | -0.4731 | 0.735052 | 0.749756 | 0.889871 | 0.811303 | 0.847772 | -0.47134 | -0.62024 | -0.4318 | -0.49979 |
| P-value | 2.33E-05 | 0.127581 | 2.47E-05 | 0.000802 | 0.120331 | 0.006457 | 0.004987 | 0.000106 | 0.001359 | 0.000496 | 0.121909 | 0.031423 | 0.161004 | 0.098017 |
